# Interpretable machine learning models for predicting the incidence of antibiotic- associated diarrhea in elderly ICU patients

**DOI:** 10.1186/s12877-024-05028-8

**Published:** 2024-05-24

**Authors:** Yating Cui, Yibo Zhou, Chao Liu, Zhi Mao, Feihu Zhou

**Affiliations:** 1 Medical School of Chinese PLA, Beijing, 100853 China; 2 Department of Critical Care Medicine, The First Medical Centre, Chinese PLA General Hospital, Beijing, 100853 China

**Keywords:** Antibiotic-associated diarrhea, ICU, Elderly, XGBoost

## Abstract

**Background:**

Antibiotic-associated diarrhea (AAD) can prolong hospitalization, increase medical costs, and even lead to higher mortality rates. Therefore, it is essential to predict the incidence of AAD in elderly intensive care unit(ICU) patients. The objective of this study was to create a prediction model that is both interpretable and generalizable for predicting the incidence of AAD in elderly ICU patients.

**Methods:**

We retrospectively analyzed data from the First Medical Center of the People’s Liberation Army General Hospital (PLAGH) in China. We utilized the machine learning model Extreme Gradient Boosting (XGBoost) and Shapley’s additive interpretation method to predict the incidence of AAD in elderly ICU patients in an interpretable manner.

**Results:**

A total of 848 adult ICU patients were eligible for this study. The XGBoost model predicted the incidence of AAD with an area under the receiver operating characteristic curve (ROC) of 0.917, sensitivity of 0.889, specificity of 0.806, accuracy of 0.870, and an F1 score of 0.780. The XGBoost model outperformed the other models, including logistic regression, support vector machine (AUC = 0.809), K-nearest neighbor algorithm (AUC = 0.872), and plain Bayes (AUC = 0.774).

**Conclusions:**

While the XGBoost model may not excel in absolute performance, it demonstrates superior predictive capabilities compared to other models in forecasting the incidence of AAD in elderly ICU patients categorized based on their characteristics.

## Background

Antibiotic-associated diarrhea (AAD) is a type of diarrhea that occurs subsequent to antibiotic administration and cannot be attributed to any other etiology. The prevalence of AAD ranges from 5 to 35% [1–3]. Critically ill patients in intensive care units (ICU) exhibit a higher incidence of AAD due to the complexity of their conditions, the diverse array of antibiotics utilized, and the frequent use of antibiotic combinations [4–6]. With the aging demographic, there has been a rise in the proportion of elderly patients in the ICU. As the elderly population experiences a reduction in beneficial commensal bacteria, the intestinal barrier becomes more vulnerable. Consequently, the elderly are more susceptible to the effects of antibiotic use, leading to an elevated risk of AAD. The occurrence of AAD prolongs hospitalization, escalates medical expenses, and may even contribute to increased mortality [7–9]. Early identification of patients at risk of AAD is critical and may facilitate timely prevention and intervention. This study aimed to construct a predictive model for AAD risk using data from the Department of Critical Care Medicine at the First Medical Center of the People’s Liberation Army General Hospital (PLAGH). The SHAP method was employed to explicate the predictive model, enabling it to not only anticipate outcomes but also provide a logical rationale for the prediction, thereby significantly bolstering user confidence in the model.

### Methods

We performed a longitudinal, monocenter, retrospective study based on PLAGH database. We reported according to the TRIPOD Checklist.

### Study population

Data on patients admitted to the Department of Critical Care Medicine at the First Medical Center of the General Hospital of the People’s Liberation Army (PLA) from January 1, 2020, to June 30, 2022, and treated with antibiotics were retrospectively analyzed. Inclusion criteria: (1) aged 60 years or older; (2) received antibiotic treatment within 7 days of admission to the ICU; (3) absence of diarrhea symptoms upon admission to the ICU. Exclusion criteria: (1) ICU length of stay ≤ 2 days; (2) palliative care; (3) diarrhea symptoms upon admission to the ICU (including previous chronic gastrointestinal diseases such as irritable bowel syndrome, ischemic bowel disease, and inflammatory bowel disease, as well as acute gastrointestinal diseases such as food poisoning, acute gastroenteritis, and laxative medication); (4) postoperative gastrointestinal tumors (i.e., admitted to the ICU with a jejunostomy, a ileostomy and a colostomy); (5) Missing clinical information.

### Grouping

Grouping was conducted based on the AAD diagnostic criteria, with individuals who met the criteria were included in the AAD group, and those who did not meet the criteria were included in the control group. The AAD group consisted of patients who met the AAD diagnostic criteria, which included the absence of diarrhea prior to admission and recent or current use of antimicrobial drugs. Symptoms of diarrhea in this context were defined as having three or more loose or watery stools per day, along with bloody or mucus-pus-blood stools, fever, abdominal pain, and other specific criteria. Other potential causes of diarrhea, such as underlying conditions and improper care, were excluded [10].

### Data extraction

We collected baseline characteristics of patients within the first 24 h of ICU admission and clinical and pharmacologic measures within 7 days of ICU admission. Demographic parameters included age, gender, and body mass index (BMI). Clinical treatment measures included mechanical ventilation, continuous renal replacement therapy (RRT), and enteral nutrition. Laboratory parameters included hemoglobin (Hb), C-reactive protein (CRP), interleukin-6 (IL-6), platelet count (Plt), procalcitonin (Pct), albumin (Alb), serum creatinine (Scr), serum phosphorus (P), amylase, and lipase. Pharmacologic interventions included third generation cephalosporin antibiotics(ceftazidime, ceftriaxone, cefoperazone sodium sulbactam sodium), carbapenem antibiotic(meropenem), glycopeptide antibiotics(ticlopidine, vancomycin), tetracycline antibiotics(tigecycline), penicillin antibiotics(piperacillin sodium tazobactam sodium), oxazolidinone antibiotics(linezolid), anti-anaerobic antibiotics(ornidazole), antifungal antibiotics(fluconazole, caspofungin) and sedative and analgesics (propofol, dexmedetomidine, midazolam, bupropion). Disease severity was assessed using the Acute Physiology and Chronic Health Evaluation II (APACHE II) [11] and Sequential Organ Failure Assessment (SOFA) score [12]. Study outcomes included the length of ICU stay and hospital mortality. The study complied with the Declaration of Helsinki and was approved by the Ethical Committee of the General Hospital of the People’s Liberation Army (PLA)(S2017-054-02). Considering that this was a retrospective observational study. Informed consent is deemed unnecessary by the Ethical Committee of the General Hospital of the People’s Liberation Army (PLA).

All computations and analyses were performed using Python version 3.9. Continuous variables were represented as means ± standard deviations (SDs) or medians and interquartile ranges (IQRs). Categorical variables were presented as totals and percentages. Group comparisons were conducted using the Kruskal-Wallis test for continuous variables, and the chi-square test and ANOVA for categorical variables. Statistical significance was defined as p-values less than 0.05. Variables with missing values exceeding 40% were excluded from further analysis, and the overall median was used to interpolate the remaining missing data. The study cohort was randomized with 70% of the data used for model training and 30% for model testing. The study employed LASSO regression analysis to identify the variables that could predict the likelihood of developing AAD. Five machine learning methods (XGBoost, Logistic Regression [LR], Support Vector Machine [SVM], k Nearest Neighbor Algorithm [KNN], and Plain Bayes [NB]) were employed to develop predictive models. Key hyperparameters of XGBoost were set to their default values, including the learning rate (learning rate = 0.1), the maximum depth of each tree (max depth = 3), and the number of modeled sequence trees (n estimators = 20). Evaluation metrics included the area under the receiver operating characteristic curve (AUC), sensitivity, specificity, accuracy, and F1 score. The F1 score combines the precision and recall of a classifier into a single score ranging from 0 to 1 [13]. Precision is defined as TP/(TP + FP) (where TP denotes true positives and FP denotes false positives), and the model’s accuracy was assessed by confirming the correct TP. Recall is defined as TP/(TP + FN) (where FN denotes false negatives) and is used to measure how many true positives are identified by the model. The F1 score is defined as 2 × (precision × recall)/(precision + recall), representing a balance between precision and recall [14, 15]. SHAP values were utilized to interpret early prediction models. They offer a unified approach for interpreting the outcomes of any machine learning model and provide consistent and locally accurate attribution values for each feature [16, 17].

## Results

### Baseline characteristics of included patients

**Fig. 1:**
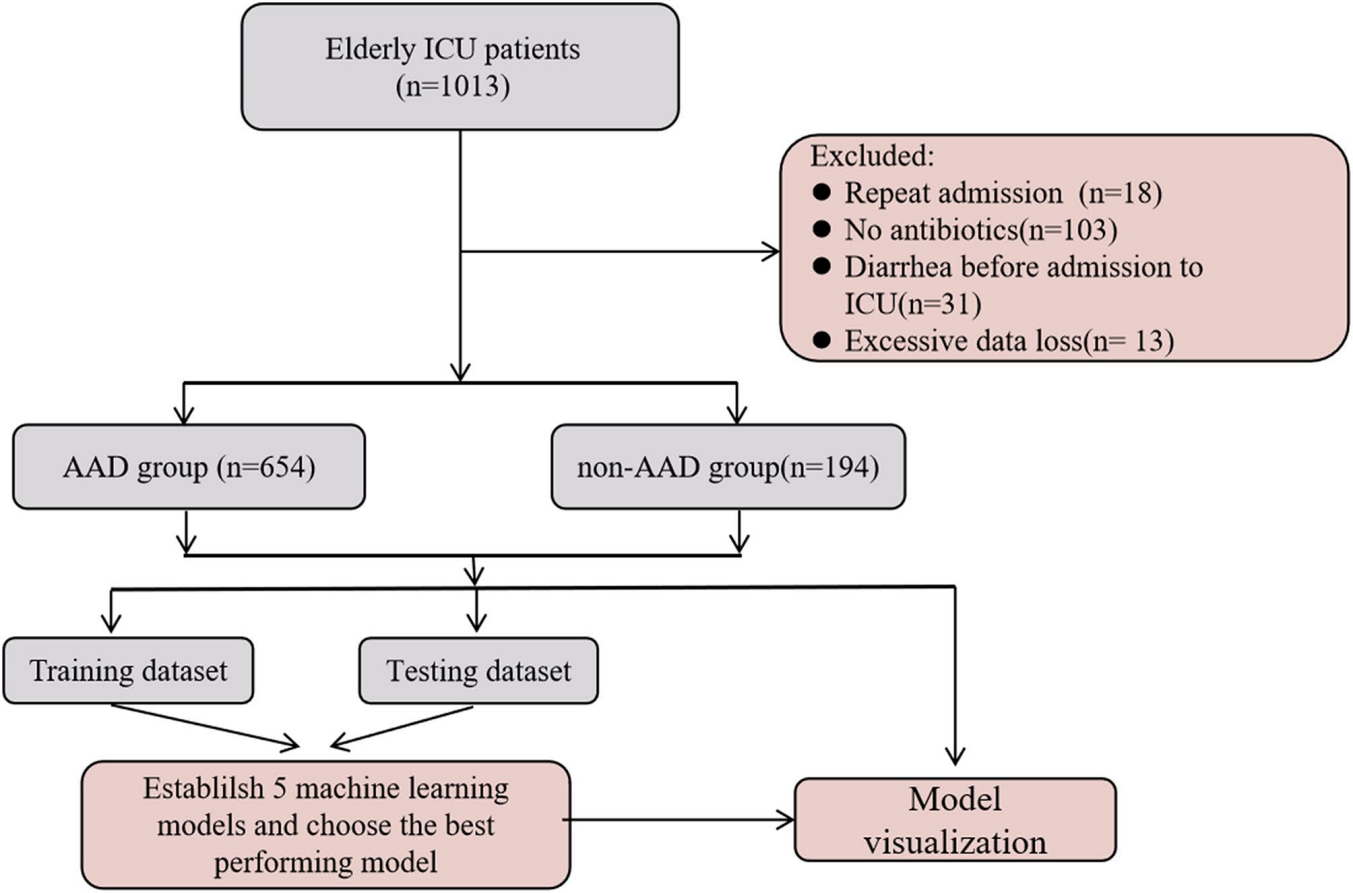
The flow chart of patient recruitment

We analyzed a total of 848 qualified adult patients for this study. The flow chart of patient recruitment is shown in Fig. 1. The dataset was split randomly into two sections: 70% of the data was utilized for training the model, and 30% was used for testing the model (Table 1). The occurrence of AAD in the training set was 22.32% (139 out of 596), and in the testing set, it was 21.82% (55 out of 252), as indicated in Table 1.

**Table 1:** Baseline characteristics of two dataests

	Training dataset(*n*=596)				Testing dataset(*n*=252)		
	Non-AAD(*n*=457)	AAD(*n*=139)	*P*-Value		Non-AAD(*n*=197)	AAD(*n*=55)	*P*-Value
**Clinical parameters**							
Male, n(%)	260 (56.9)	83 (59.7)	0.623		108 (54.8)	32 (58.2)	0.772
age(years)	73.0 (66.0-81.0)	74.0 (67.5-82.5)	0.098		74.0 (68.0-82.0)	75.0 (67.5-82.0)	0.801
BMI	23.8 (21.3-25.6)	23.8 (22.6-25.0)	0.335		23.2 (20.8-24.6)	23.8 (21.0-25.6)	0.258
**Therapeutic measures**							
Mechanical ventilation, n(%)	374 (81.8)	93 (66.9)	<0.001		156 (79.2)	38 (69.1)	0.164
RRT, n(%)	31 (6.8)	23 (16.5)	0.001		10 (5.1)	14 (25.5)	<0.001
Enteral nutrition, n(%)	137 (30.0)	103 (74.1)	<0.001		59 (29.9)	39 (70.9)	<0.001
**Laboratory parameters**							
WBC(10^9/L)	9.4 (7.2-12.6)	10.2 (7.4-14.0)	0.375		9.9 (6.9-13.1)	10.5 (7.8-13.7)	0.193
Hemoglobin(g/L)	107.0 (92.0-122.0)	95.0 (83.5-109.0)	<0.001		103.0 (91.0-116.0)	93.0 (83.5-109.0)	0.008
C-reactive protein(mg/L)	1.2 (0.2-3.9)	3.5 (1.3-7.9)	<0.001		1.4 (0.3-4.2)	3.9 (1.6-8.9)	0.001
Interleukin-6 (pg/ml)	78.3 (33.0-217.7)	90.0 (27.2-230.7)	0.911		110.4 (40.0-291.3)	94.4 (43.0-252.2)	0.761
Platelet(10^9/L)	172.0 (127.0-224.0)	158.0 (99.5-230.5)	0.147		175.0 (131.0-231.0)	156.0 (125.5-231.0)	0.369
Procalcitonin (ng/ml)	0.1 (0.1-0.6)	0.8 (0.2-2.2)	<0.001		0.1 (0.1-0.7)	1.2 (0.2-3.2)	<0.001
Albumin(g/L)	31.3 (27.2-34.1)	31.0 (27.6-33.8)	0.634		29.9 (25.6-34.0)	30.6 (27.4-33.9)	0.285
Serum creatinine(μmol/L)	72.0 (54.9-92.1)	80.2 (57.8-106.6)	0.035		69.8 (56.0-87.4)	88.1 (66.0-118.6)	0.001
P(mmol/L)	1.2 (0.9-1.4)	1.0 (0.7-1.3)	<0.001		1.1 (0.9-1.4)	1.0 (0.8-1.3)	0.095
Lipase(U/L)	22.7 (15.0-39.6)	36.4 (17.2-64.4)	<0.001		21.8 (13.8-38.2)	26.8 (17.8-56.7)	0.022
**Severity of illness**							
CCI	4.0 (4.0-6.0)	4.0 (4.0-5.0)	0.851		4.0 (4.0-6.0)	4.0 (4.0-6.0)	0.649
APACHE II	18.0 (12.0-20.0)	17.0 (11.0-21.0)	0.994		17.0 (13.0-20.0)	18.0 (12.5-21.0)	0.551
SOFA	5.0 (3.0-6.0)	5.0 (4.0-8.0)	0.019		5.0 (4.0-6.0)	6.0 (4.0-7.0)	0.062
**Medications**							
Ceftazidime, n (%)	137 (30.0)	37 (26.6)	0.512		46 (23.4)	10 (18.2)	0.528
Ceftriaxone, n (%)	22 (4.8)	10 (7.2)	0.381		5 (2.5)	2 (3.6)	0.649
Meropenem, n (%)	69 (15.1)	41 (29.5)	<0.001		34 (17.3)	20 (36.4)	0.004
Teicoplanin, n (%)	176 (38.5)	37 (26.6)	0.014		76 (38.6)	21 (38.2)	1
Tigecycline, n (%)	7 (1.5)	7 (5.0)	0.025		1 (0.5)	5 (9.1)	0.002
Cefoperazone/sulbactam, n (%)	109 (23.9)	36 (25.9)	0.704		48 (24.4)	11 (20.0)	0.62
Piperacillin/Tazobactam, n (%)	54 (11.8)	25 (18.0)	0.083		18 (9.1)	8 (14.5)	0.36
Flomoxef, n (%)	73 (16.0)	12 (8.6)	0.042		40 (20.3)	3 (5.5)	0.017
Biapenem, n (%)	56 (12.3)	15 (10.8)	0.752		34 (17.3)	8 (14.5)	0.785
Linezolid, n (%)	21 (4.6)	22 (15.8)	<0.001		12 (6.1)	8 (14.5)	0.05
Vancomycin, n (%)	38 (8.3)	26 (18.7)	0.001		8 (4.1)	6 (10.9)	0.087
Fluconazole, n (%)	29 (6.3)	26 (18.7)	<0.001		12 (6.1)	12 (21.8)	0.001
Caspofungin, n (%)	11 (2.4)	13 (9.4)	0.001		7 (3.6)	7 (12.7)	0.016
Propofol, n (%)	335 (73.3)	80 (57.6)	0.001		146 (74.1)	32 (58.2)	0.033
Dexmedetomidine, n (%)	236 (51.6)	47 (33.8)	<0.001		90 (45.7)	25 (45.5)	1
Midazolam, n (%)	187 (40.9)	62 (44.6)	0.501		81 (41.1)	26 (47.3)	0.508
Butorphanol, n (%)	366 (80.1)	81 (58.3)	<0.001		160 (81.2)	39 (70.9)	0.141
Remifentanil, n (%)	69 (15.1)	21 (15.1)	1.000		21 (10.7)	9 (16.4)	0.358
**Outcomes**							
ICU mortality, n (%)	18 (3.9)	17 (12.2)	0.001		10 (5.1)	7 (12.7)	0.064
Length of ICU stay(d)	3.8 (2.8-6.0)	13.0 (5.8-27.5)	<0.001		3.7 (2.7-6.1)	15.0 (5.9-36.5)	<0.001

AAD, Antibiotic associated diarrhea; RRT, Renal replacement therapy; CCI, Charlson Comorbidity Index; WBC, white blood cell; APACHE II, Acute Physiology and Chronic Health Evaluation; SOFA, Sequential Organ Failure Assessment; ICU, Intensive care units

### Modeling

**Fig. 2:**
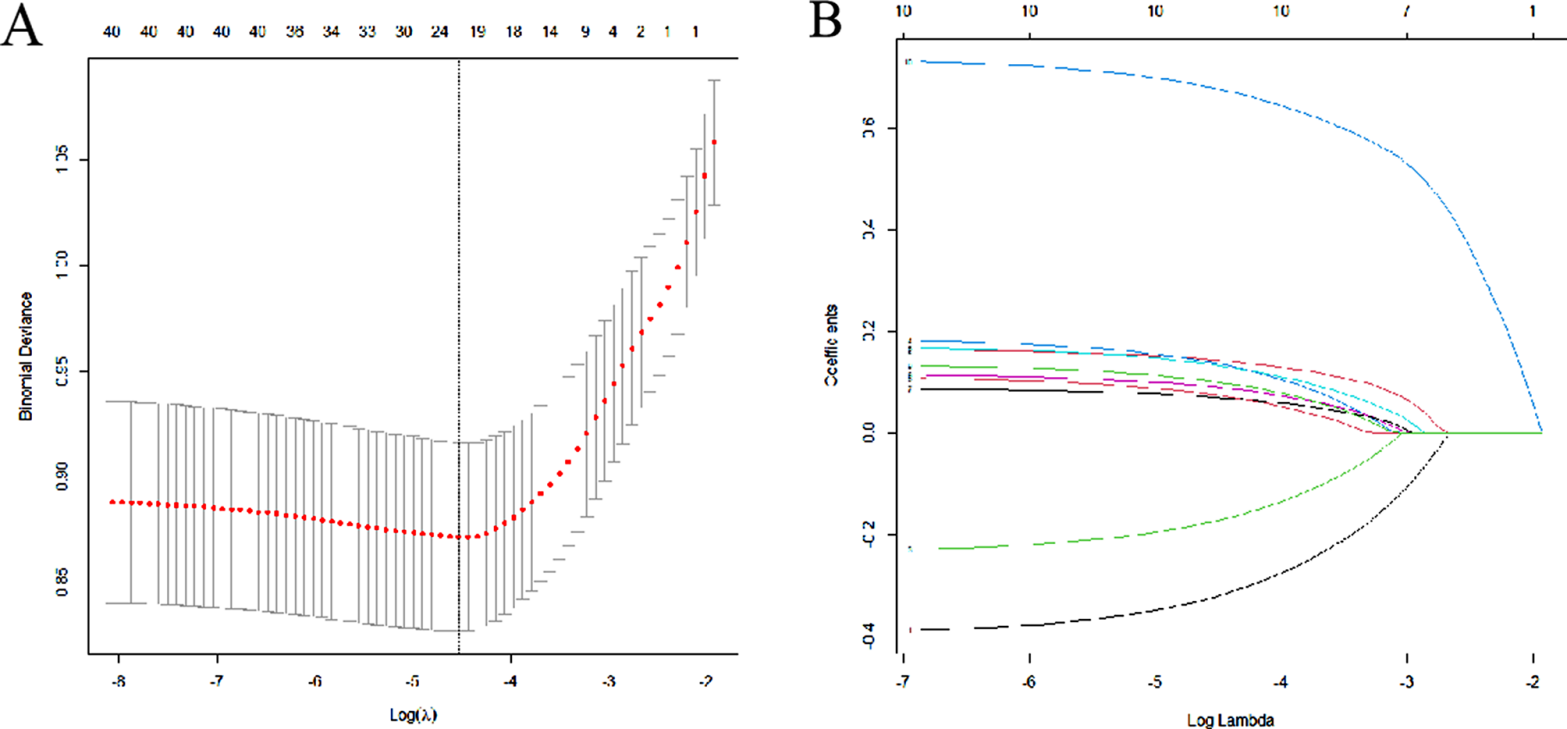
Variable selection using the least absolute shrinkage and selection operator (LASSO) binary logistic regression model. (A) LASSO coefficient profiles of the 37 baseline features. (B) Tuning parameter (λ) selection in the LASSO model used 5-fold cross-validation via minimum criteria variable selection

**Fig. 3:**
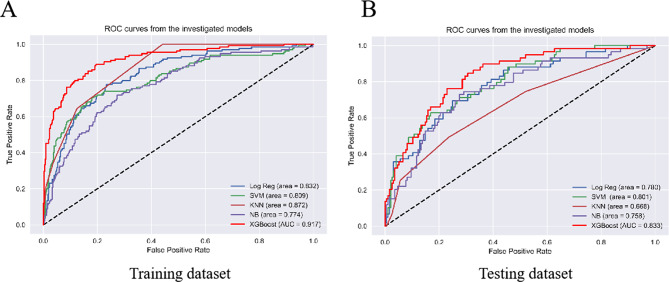
Predictive performance of training dataset(A) and testing dataset(B) evaluated by machine learning methods. Log Reg, logistic regression; SVM, support vector machine; KNN, k-nearest neighbor; Tree, Decision Tree; NB, naive Bayesian; XGBoost, extreme gradient boosting

37 variables measured at admission were included in the Lasso regression analysis. After Lasso regression selection (see Fig. 2). 10 variables were identified as predictors of AAD occurrence. These variables included hemoglobin, C-reactive protein, use of tigecycline, butorphanol, vancomycin, linezolid, fluconazole, meropenem, enteral nutrition, and renal replacement therapy. We employed various machine learning techniques, including XGBoost, LR, SVM, KNN and NB, to predict the occurrence of AAD in elderly ICU patients using all available variables as input features. The findings revealed that XGBoost achieved the highest AUC for the test dataset (0.917, 95% confidence interval = 0.881–0.948) (Fig. 3; Table 2).

**Table 2:** The performance of each model for prediction

Model	AUC (%)	SE (%)	SP (%)	AC (%)	F1 score
Log Reg	0.832	0.778	0.766	0.766	0.718
SVM	0.809	0.704	0.821	0.833	0.710
KNN	0.872	1.000	0.559	0.823	0.683
NB	0.774	0.719	0.727	0.759	0.683
XGBoost	0.917	0.889	0.806	0.870	0.780

SE, sensitivity; SP, specificity; AC, accuracy. Log Reg, logistic regression; SVM, support vector machine; KNN, k-nearest neighbor; NB, naive Bayesian; XGBoost, extreme gradient boosting

### Model evaluation

**Fig. 4:**
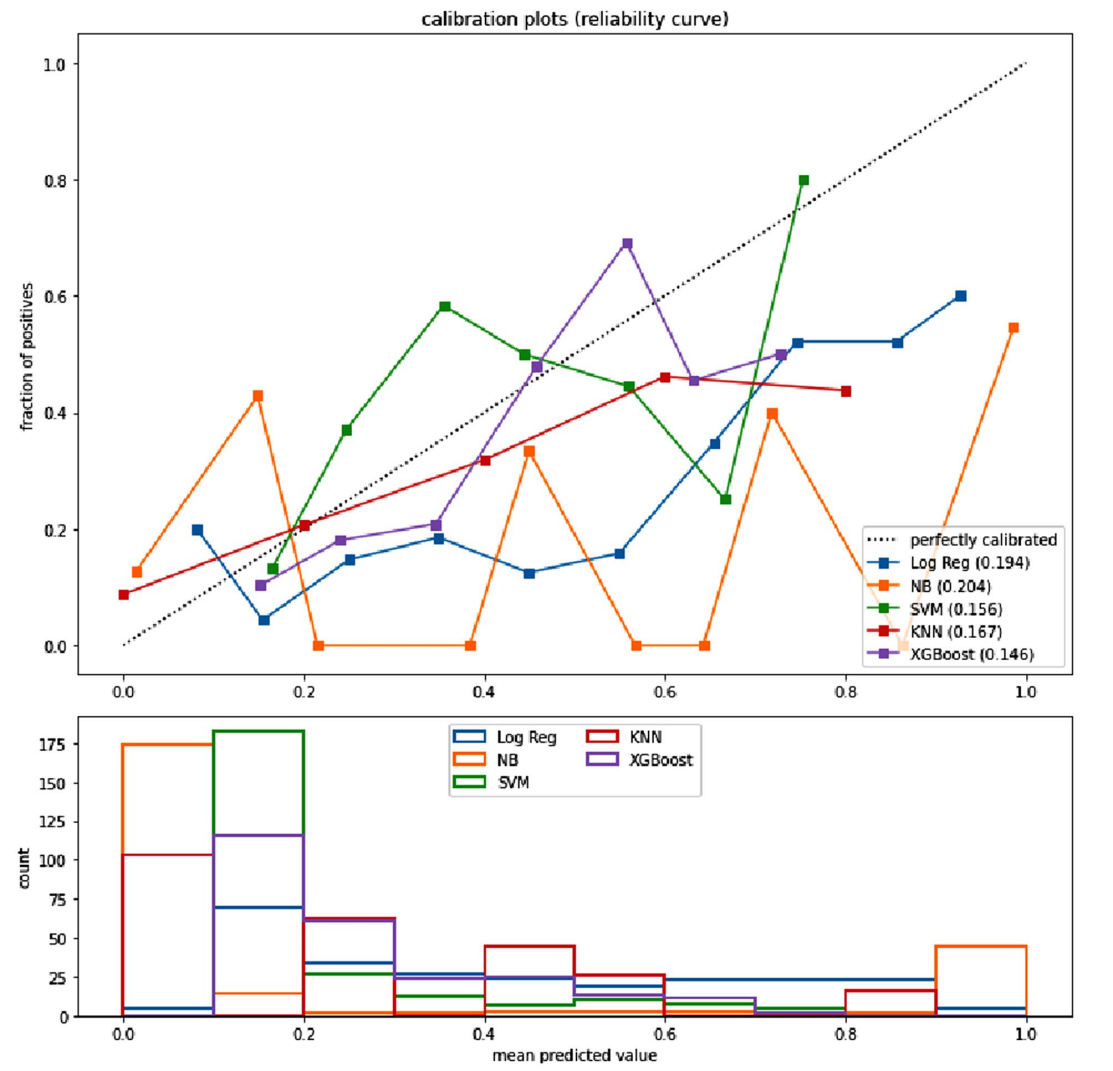
Calibration plots of five models. The XGBoost achieved lower(better) Brier scores(0.146) compared with the other models

**Fig. 5:**
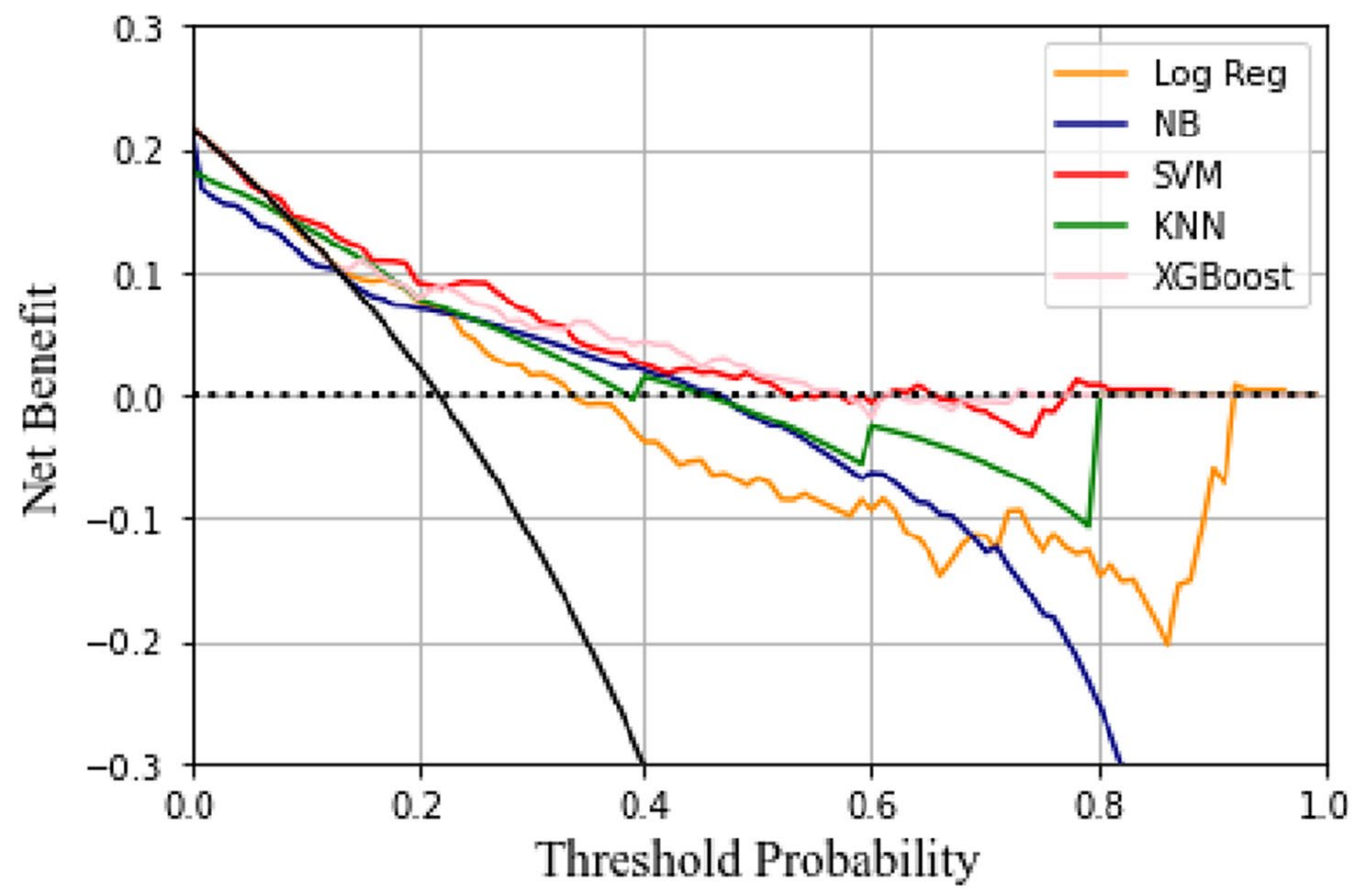
Decision curve analysis for five machine learnin models. The XGBoost model can serve as the best prediction to AAD

Brier scores and DCA are important metrics used to evaluate predictive models. XGBoost’s Brier score is much lower and better than other models. Figure 4 shows the calibration curves for the nine models. The DCA shows that the XGBoost model can be used as a tool to predict the occurrence of AAD (Fig. 5). We also applied K-fold cross-validation to evaluate the model performance, as shown in Table 3.

**Table 3:** The K-fold crossing-validation of each model for prediction

Model	Average of crossing-validation score	Standard error of crossing-validation score
Log Reg	0.666	0.113
SVM	0.766	0.088
KNN	0.786	0.031
NB	0.782	0.068
XGBoost	0.810	0.030

Log Reg, logistic regression; SVM, support vector machine; KNN, k-nearest neighbor; NB, naive Bayesian; XGBoost, extreme gradient boosting

### Model interpretation

**Fig. 6:**
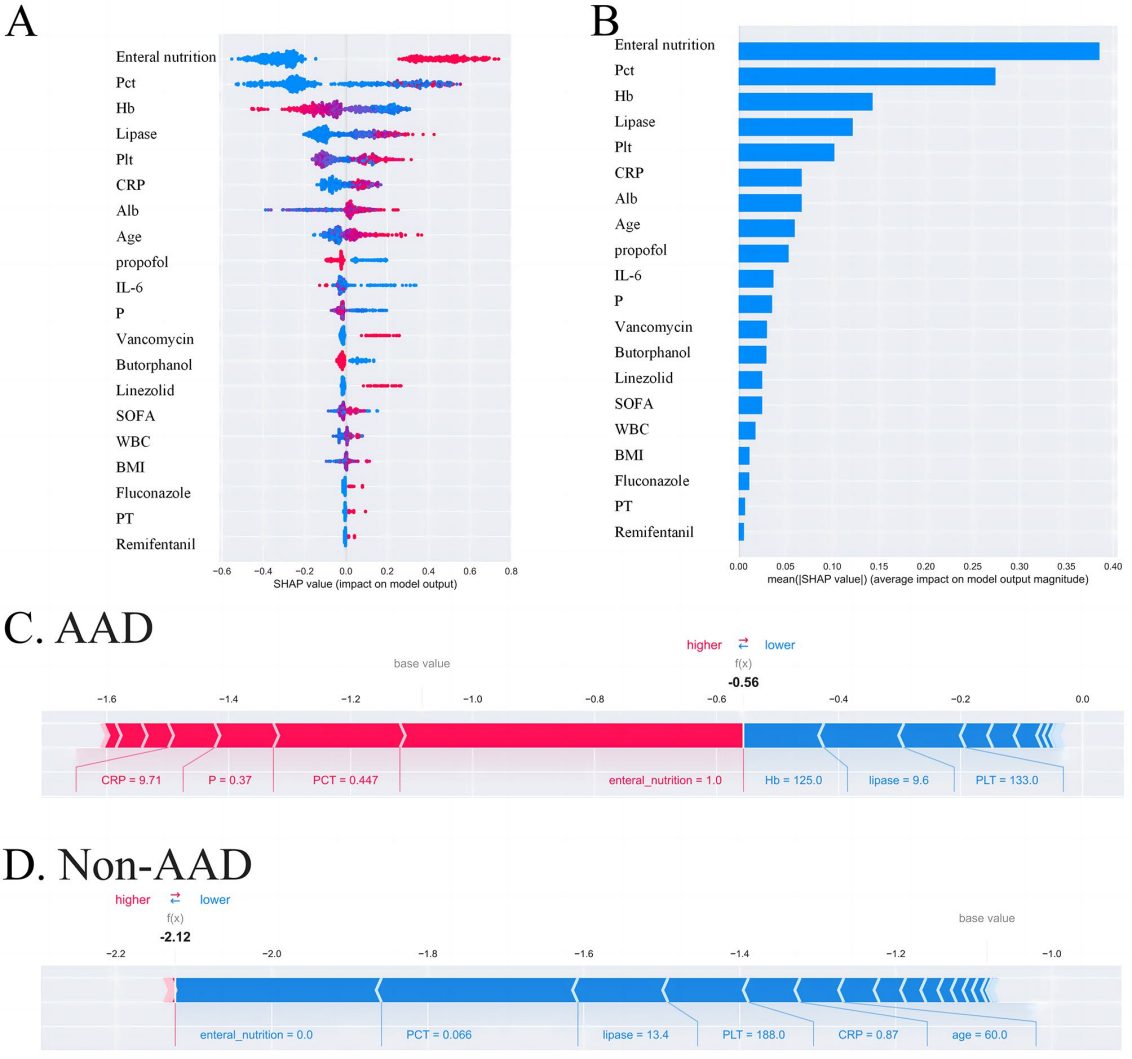
The model’s interpretation. A, The importance ranking of the top 20 risk factors. The SHAP value (x-axis) is a unified index responding to the effect of a feature in the model. In each feature importance row, all patients’ attributes to the outcome were plotted using different colored dots, in which the red (blue) dots represent high (low) values. The higher the SHAP value of a feature, the higher the risk of death for the patient. B, The importance ranking of the top 20 variables according to the mean (|SHAP value|). C, D, The interpretation of model prediction results with the two samples. This explanation shows features each contributing to pushing the model output from the base value to the model output. Features pushing the prediction higher are shown in red, and those pushing the prediction lower are shown in blue. C, The AAD patient was predicted to occur AAD because of their high C-reaction protein(CRP)(9.71 mg·L − 1) level, high serium phosphorus(P)(0.37 mmol·L − 1) level, high procalcitonin(PCT)(0.447ng· mL − 1) level and the use of enteral nutrition; D, The non-AAD patient was predicted to be normal defecation function because of a lower procalcitonin (0.066ng· mL − 1) level, lower lipase (13.4U·L − 1) level, normal platelet(PLT)(188*10^9 ·L − 1) level, lower C-reaction protein(CRP)(0.87 mg·L − 1) level, lower age(60y). WBC, white blood cell; Pct, procalcitonin; Alb, albumin; Hb, hemoglobin; IL-6, interleukin-6; Scr, serum creatinine; Plt, platelet; CRP, C-reaction protein; SOFA, Sequential Organ Failure Assessment; BMI, body mass index; CCI, Charlson comorbidity index; PT, Piperacillin/Tazobactam

**Fig. 7:**
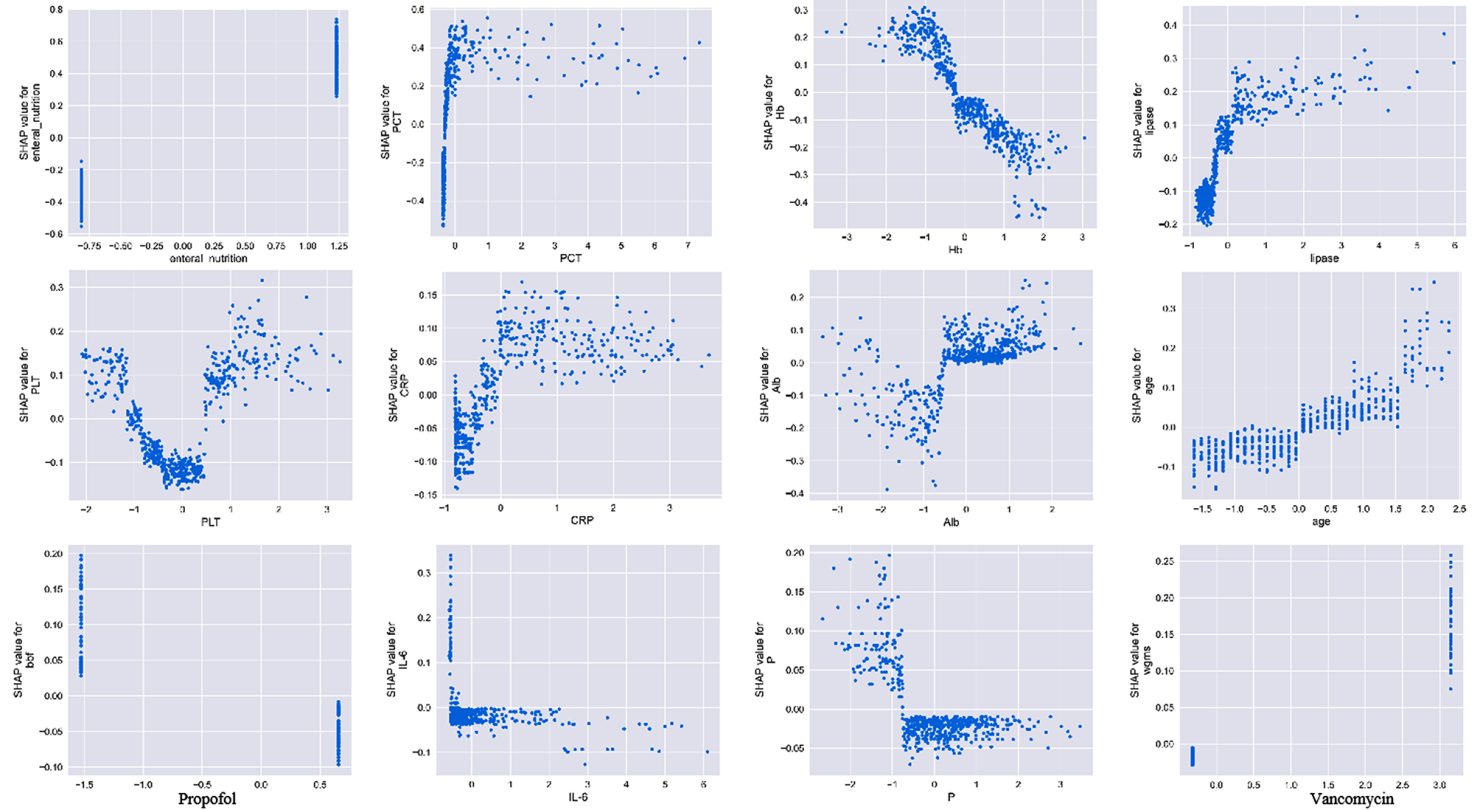
SHAP dependence plot of the XGboost model. The SHAP dependence plot shows how a single feature affects the output of the XGBoost prediction model. SHAP values for specific features exceed zero. WBC, white blood cell; Pct, procalcitonin; Alb, albumin; Hb, hemoglobin; IL-6, interleukin-6; Scr, serum creatinine; Plt, platelet; CRP, C-reaction protein; SOFA, Sequential Organ Failure Assessment; BMI, body mass index; CCI, Charlson comorbidity index; PT, Piperacillin/Tazobactam

Based on the above, we can conclude that the XGBoost model significantly outperforms the other four machine learning models. Therefore, we apply the SHAP model to explain the XGBoost model. The diagram in Fig. 6 illustrates the ranking of the top 20 risk factors and their importance. The SHAP value, represented on the x-axis, acts as a standardized measure of a feature’s impact on the response model. Each row in the feature importance chart displays patient attributes related to the outcome using different colored dots, with red and blue dots indicating high and low values, respectively. A higher SHAP value for a characteristic indicates a greater risk of patient morbidity. The first 20 variables are presented in descending order of mean importance (SHAP value). Additionally, the model prediction results are interpreted based on two samples from the dataset. This interpretation highlights the features contributing to pushing the model output away from the base value. Features that increase the prediction are depicted in red, while those that decrease the prediction are shown in blue. For instance, the high risk of acute aortic dissection (AAD) in patients was predicted to be associated with elevated levels of c-reactive protein (CRP) (9.71 mg/L), high serum phosphorus (P) levels (0.37 mmol/L), elevated procalcitonin (PCT) levels (0.447 ng/mL), and the use of enteral nutrition. Conversely, non-AAD patients were predicted to have lower levels of calcitoninogen (0.066 ng/mL), lower levels of adiponectin (13.4 U/L), normal platelet levels (PLT) (188 × 10^9/L), lower levels of C-reactive protein (CRP) (0.87 mg/L), and a younger age (60 years). Figure 7 presents the SHAP dependency plot for the top 12 important variables. It was observed that elevated levels of calcitoninogen, interleukin-6, adiponectin, and C-reactive protein, as well as older age, vancomycin use, and enteral nutrition, were associated with a higher incidence of AAD. Conversely, lower levels of hemoglobin, serum phosphorus, and platelets were linked to a higher incidence of AAD. The use of the sedative propofol may reduce the incidence of AAD in elderly ICU patients. Finally, the confusion matrix was utilized to display the prediction outcomes of the XGBoost model, with a positive predictive value of 84.6% and a negative predictive value of 86.6%.

## Discussion

In this study, we developed and internally validated a machine learning algorithm using 37 features to predict the occurrence of AAD in elderly patients in the ICU. The XGBoost model outperforms LR, SVM, KNN and NB. The variables necessary for calculating the risk of AAD occurrence are typically readily available at the time of admission. Additionally, we have employed SHAP to interpret the XGBoost model, which will aid physicians in comprehending the decision-making process of the model. Early and aggressive preventive measures are imperative if a patient is at high risk of developing AAD.

The widespread use of broad-spectrum antibiotics in recent years has raised global concerns about the incidence of AAD in elderly ICU patients [18]. The occurrence of AAD in patients can extend the length of hospital stay, raise healthcare expenses, and potentially contribute to higher mortality [10, 19, 20]. As a result, it is essential to prevent the occurrence of AAD and to identify and treat it as early as possible. Most current studies focus on analyzing the risk factors for AAD in ICU patients. For example, a retrospective study conducted at a single center analyzed the risk factors for AAD in ICU patients. The study found that advanced age, prolonged ICU stay, extended use of proton pump inhibitors, and prolonged antibiotic use were associated with a higher risk of AAD in elderly ICU patients [21]。No study has developed a model to predict the risk of AAD in elderly ICU patients. In the current study, we have developed a predictive model incorporating 40 characteristics based on the PLAGH data. The top 20 risk factors with the highest predictive value were enteral nutrition use, calcitoninogen, hemoglobin, lipase, platelets, C-reactive protein, albumin, age, propofol use, interleukin-6, serum phosphorus, vancomycin use, butorphanol use, linezolid use, SOFA score, white blood cell count, body mass index, fluconazole use, piperacillin sodium tazobactam sodium use, and remifentanil use. The SHAP method was also utilized to interpret the predictive model, enabling it to not only forecast the user’s expected outcome but also to offer a rational explanation for the prediction. This significantly enhanced the user’s trust in the model.

In our investigation, we observed that the administration of sedative and analgesic medications (specifically propofol, butorphanol, and remifentanil) was associated with a decreased risk of AAD. This reduction is likely attributed to the inhibitory effects of opioids on gastrointestinal motility, resulting in reduced bowel movements [22, 23]. This decrease may lead to disturbances in the gut microbiota and intestinal barrier function, thereby increasing the likelihood of bacterial translocation [24]. However, it is important to note that the overall impact may not necessarily be a protective factor. Previous research has indicated that nearly all classes of antibiotics may contribute to the onset of AAD [25, 26].

A retrospective analysis revealed that cefoperazone/sulbactam or piperacillin/ tazobactam resulted in a similar incidence of AAD [27]. Nevertheless, there is a lack of comparative studies examining the effects of different antibiotics on AAD incidence. The antibiotics identified as the top 20 risk factors with the highest predictive value in our study were vancomycin, linezolid, fluconazole, and piperacillin sodium-tazobactam sodium. Among these, vancomycin exhibited the most significant impact in predicting the occurrence of AAD in elderly ICU patients.

The study has a few potential limitations. Firstly, it employed a small sample size for model development and lacked an external validation cohort. Consequently, future multicenter studies with larger sample sizes are imperative to assess the model’s generalizability. Secondly, the medication therapy considered in the training and testing datasets only encompassed antibiotics and sedative-analgesic medications, neglecting other medications that could significantly influence the incidence of AAD. This oversight may limit the model’s applicability.

## Conclusion

In summary, we developed five different AAD prediction models and calibrated them using AUROC, Brier Score, and DCA to select the best performing model. The best machine learning algorithm with good performance is selected. We hope that this model can aid physicians in early intervention and treatment, potentially reducing the length of ICU hospitalization and healthcare costs for elderly patients.

## Data Availability

The datasets utilized and/or examined in the present study can be obtained from the corresponding author upon request. Email: feihuzhou301@126.com.

## Abbreviations

AAD: Antibiotic-associated diarrhea
PLAGH: People’s Liberation Army General Hospital
ICU: Intensive care unit
XGBoost: Extreme gradient boosting
AUC: Area under the receiver operating characteristic curve
SOFA: Sequential Organ Failure Assessment
APACHE II: Acute Physiology and Chronic Health Evaluation II
CRP: C-reactive protein
P: Phosphorus
PCT: Procalcitonin
PLT: Platelet levels
BMI: Body mass index
RRT: Continuous renal replacement therapy
Hb: Hemoglobin
IL-6: Interleukin-6
Alb: Albumin
Scr: Serum creatinine
SDs: Standard deviations
IQRs: Interquartile ranges
LR: Logistic Regression
SVM: Support Vector Machine
KNN: K Nearest Neighbor Algorithm
NB: Plain Bayes
TP: True positives
FP: False positives

## References

[1] Mekonnen SA, Merenstein D, Fraser CM, Marco ML (2020). Molecular mechanisms of probiotic prevention of antibiotic-associated diarrhea. Curr Opin Biotechnol.

[2] Jingjing S, Yanshu Z, Yu L, Qindong S, Xue W, Lei Z (2019). Factors related to antibiotic-associated diarrhea in patients in the intensive care unit receiving antifungals: a single-center retrospective study. J Int Med Res.

[3] Motamedi H, Fathollahi M, Abiri R, Kadivarian S, Rostamian M, Alvandi A (2021). A worldwide systematic review and meta-analysis of bacteria related to antibiotic-associated diarrhea in hospitalized patients. PLoS ONE.

[4] Dai M, Liu Y, Chen W, Buch H, Shan Y, Chang L (2019). Rescue fecal microbiota transplantation for antibiotic-associated diarrhea in critically ill patients. Crit Care.

[5] Ghoshal UC, Gwee KA, Holtmann G, Li Y, Park SJ, Simadibrata M (2021). Physician perceptions on the use of antibiotics and probiotics in adults: an International Survey in the Asia-Pacific Area. Front Cell Infect Microbiol.

[6] Guo X, Sun L, Wang S, Shen Y. Effects of Irrational Use of antibiotics on Intestinal Health of Children with Extraintestinal Infectious diseases. Volume 2022. Contrast media & molecular imaging; 2022. p. 9506490. 10.1155/2022/9506490 PMC9410831 36051926

[7] Lau VI, Xie F, Fowler RA, Rochwerg B, Johnstone J, Lauzier F (2022). Health economic evaluation alongside the Probiotics to prevent severe pneumonia and endotracheal colonization trial (E-PROSPECT): a cost-effectiveness analysis. Can J Anaesth = J Canadien D’anesthesie.

[8] Zhang L, Zeng X, Guo D, Zou Y, Gan H, Huang X (2022). Early use of probiotics might prevent antibiotic-associated diarrhea in elderly (> 65 years): a systematic review and meta-analysis. BMC Geriatr.

[9] Zhao L, Zhang Y, Wang Y, Qiao H, Wang Y, Ren J (2023). Gut microbiota diversity of hospitalized older adult patients with and without antibiotic-associated diarrhea. Aging Clin Exp Res.

[10] Rajkumar C, Wilks M, Islam J, Ali K, Raftery J, Davies KA (2020). Do probiotics prevent antibiotic-associated diarrhoea? Results of a multicentre randomized placebo-controlled trial. J Hosp Infect.

[11] Knaus WA, Wagner DP, Draper EA, Zimmerman JE, Bergner M, Bastos PG (1991). The APACHE III prognostic system. Risk prediction of hospital mortality for critically ill hospitalized adults. Chest.

[12] Vincent JL, Moreno R, Takala J, Willatts S, De Mendonca A, Bruining H (1996). The SOFA (Sepsis-related Organ failure Assessment) score to describe organ dysfunction/failure. On behalf of the Working Group on Sepsis-related problems of the European Society of Intensive Care Medicine. Intensive Care Med.

[13] Handelman GS, Kok HK, Chandra RV, Razavi AH, Lee MJ, Asadi H (2018). eDoctor: machine learning and the future of medicine. J Intern Med.

[14] Greener JG, Kandathil SM, Moffat L, Jones DT (2022). A guide to machine learning for biologists. Nat Rev Mol Cell Biol.

[15] Choi RY, Coyner AS, Kalpathy-Cramer J, Chiang MF, Campbell JP (2020). Introduction to machine learning, neural networks, and Deep Learning. Translational Vis Sci Technol.

[16] Belle V, Papantonis I (2021). Principles and practice of Explainable Machine Learning. Front big data.

[17] Wang Z, Zhang L, Huang T, Yang R, Cheng H, Wang H (2023). Developing an explainable machine learning model to predict the mechanical ventilation duration of patients with ARDS in intensive care units. Heart lung: J Crit care.

[18] Velasco M, Requena T, Delgado-Iribarren A, Peláez C, Guijarro C (2019). Probiotic yogurt for the Prevention of Antibiotic-associated diarrhea in adults: a Randomized double-blind placebo-controlled trial. J Clin Gastroenterol.

[19] Wright K, Wright H, Murray M (2015). Probiotic treatment for the prevention of antibiotic-associated diarrhoea in geriatric patients: a multicentre randomised controlled pilot study. Australas J Ageing.

[20] Zhang Y, Sun J, Zhang J, Liu Y, Guo L (2018). Enzyme inhibitor antibiotics and antibiotic-Associated Diarrhea in critically ill patients. Med Sci Monitor: Int Med J Experimental Clin Res.

[21] Zhou H, Xu Q, Liu Y, Guo LT (2020). Risk factors, incidence, and morbidity associated with antibiotic-associated diarrhea in intensive care unit patients receiving antibiotic monotherapy. World J Clin Cases.

[22] Brenner DM, Argoff CE, Fox SM, Bochenek W, D’Astoli P, Blakesley RE (2020). Efficacy and safety of linaclotide for opioid-induced constipation in patients with chronic noncancer pain syndromes from a phase 2 randomized study. Pain.

[23] Dionne JC, Johnstone J, Smith O, Rose L, Oczkowski S, Arabi Y (2020). Content analysis of bowel protocols for the management of constipation in adult critically ill patients. J Crit Care.

[24] Kolli U, Jalodia R, Moidunny S, Singh PK, Ban Y, Tao J (2023). Multi-omics analysis revealing the interplay between gut microbiome and the host following opioid use. Gut Microbes.

[25] Soki J, Wybo I, Wirth R, Hajdu E, Matuz M, Burian K (2022). A comparison of the antimicrobial resistance of fecal Bacteroides isolates and assessment of the composition of the intestinal microbiotas of carbapenem-treated and non-treated persons from Belgium and Hungary. Anaerobe.

[26] Hagihara M, Kuroki Y, Ariyoshi T, Higashi S, Fukuda K, Yamashita R (2020). Clostridium butyricum modulates the Microbiome to protect intestinal barrier function in mice with Antibiotic-Induced Dysbiosis. iScience.

[27] Chen Y, Xiang Q, Liu L (2021). Comparison of antibiotic-associated diarrhea caused by cefoperazone/sulbactam or piperacillin/tazobactam in neurosurgery patients. J Int Med Res.

